# Mass Spectrometric Study of NF_2_, NF_3_, N_2_F_2_, and N_2_F_4_

**DOI:** 10.6028/jres.065A.041

**Published:** 1961-10-01

**Authors:** John T. Herron, Vernon H. Dibeler

## Abstract

Appearance potentia’s have been measured for selected ions from NF_2_, NF_3_, N_2_F_2_, and N_2_F_4_. Ionization-dissociation processes are identified and bond dissociation energies are calculated. In addition, the bond dissociation energy, D(F_2_N–NF_2_), has been directly measured to be 5.14±0.38 kj/mole (21.5± 1.6 kcal/mole). A summary is made of available thermochemical and mass spectrometric data for N–F compounds and some evidence is presented to support the designation of *cis* and *trans* structures for the N_2_F_2_ isomers.

## 1. Introduction

The synthesis of a new series of compounds containing nitrogen and fluorine atoms has aroused considerable interest in their chemical and physical properties; in particular, heats of formation, bond dissociation energies, and ionization processes. Some of these data have been obtained from mass spectrometric studies [1, 2, 3].^1^ In general, however, the data are fragmentary and in some cases are based on doubtful assumptions by analogy to N—H compounds. In a previous paper [3], we reported an electron impact study of tetrafluorohydrazine in which a value of 53 kcal/mole for the F_2_N–NF_2_ bond dissociation energy was calculated from estimated values of the N–F bonds in NF_3_ [1]. It was also suggested that the failure of other workers to find ions of *m*/*e* greater than that corresponding to NF_2_^+^ in the mass spectrum of N_2_F_4_ was due to decomposition of N_2_F_4_ into NF_2_ radicals in the mass spectrometer ion source. In light of the recently reported [4] value of 19.2 kcal/mole for the dissociation energy of the N–N bond in N_2_F_4_ this seems quite reasonable. We have made measurements of the effect of temperature on the N_2_F_4_^+^/NF_2_^+^ ratio in the mass spectrum of N_2_F_4_. In addition, we have made a mass spectrometric study of the thermal dissociation of N_2_F_4_, and re-examined the ionization-dissociation processes for this molecule. We report appearance potentials of various ions in the related N—F compounds: NF_2_, NF_3_, and the two available isomers of N_2_F_2_.

A recent study [5] of the absorption spectra of the N_2_F_2_ isomers has given rise to a controversy concerning their structure. Although not unequivocal, the data reported here give evidence for the similarity in bond energies and heats of formation of these isomers and hence support the designation of the N_2_F_2_ isomers as *cis* and *trans*.

## 2. Experimental Procedure

The mass spectrometer used in this research is a first order, direction focusing instrument with a nominal 60° sector field and a 12-in. radius of curvature. The analyser tube and the source and collector housings are fabricated from nonmagnetic stainless steels and made vacuum tight with gold wire gaskets. Separate pumping systems are provided for the source housing and analyser tube. The source housing contains a flanged re-entrant port to admit thermal reactors or electrodeless discharge tubes for the introduction of free radicals or other active species to the ion source with a minimum of wall collisions. In addition, the electron impact source is provided with a conventional gas introduction system.

Carefully regulated power supplies are utilized for the magnet current, the ion accelerating voltage and focusing controls and the electron emission circuit. The latter circuit is designed to permit the precise measurement of appearance potentials of either positive or negative ions and to examine ionization probability curves over the range from zero to 100 ev.

The resolved ion currents are detected by means of a 14-stage electron multiplier. The integrated ion current is measured with a vibrating-reed electrometer and pen recorder. The nominal detection limit for this system was about 10^−17^ amps.

**Figure 1:**
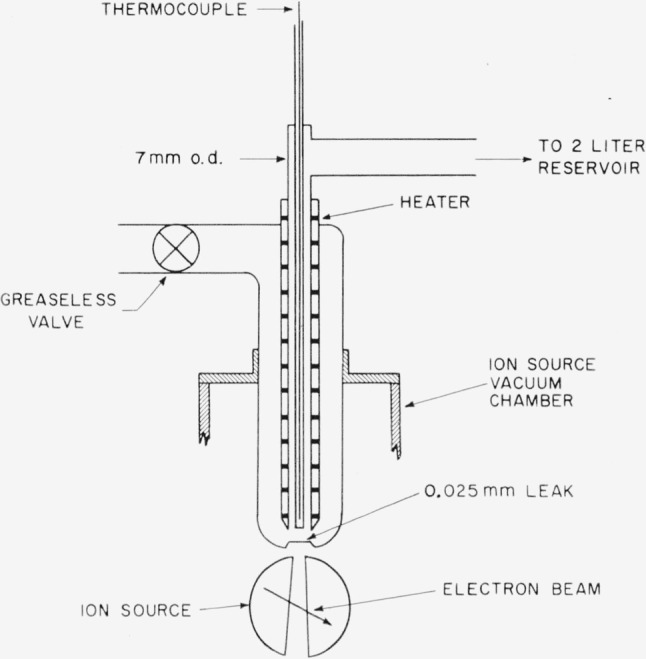
Thermal reactor for kinetic studies of the dissociation of *N*_2_*F*_4_

A simple thermal reactor was attached to the mass spectrometer to study the dissociation of N_2_F_4_. The reactor, shown schematically in figure 1, was connected to a 2-liter reservoir volume which remained at room temperature. The N_2_F_4_ at a pressure of about 0.2 mm effused from the reactor through a 1-mil glass leak located at the line-of-sight inlet to the ion source. The temperature of the N_2_F_4_ vapor was measured by a glass-encased thermocouple located about 1 mm from the leak.

The temperature variation of the mass spectrum of N_2_F_4_ was studied using the technique described by Reese, Dibeler, and Mohler [6]. Briefly, the mass spectrometer filament is turned off and the ion source allowed to cool to room temperature. The N_2_F_4_ at normal operating pressures is admitted to the ion source through the conventional gas inlet and the filament turned on. Ion currents for the NF_2_^+^ and N_2_F_4_^+^ ions were measured immediately and remeasured at frequent intervals using nominal 70 ev electron energies. The temperature was monitored by means of a thermocouple attached directly to the ion source.

Appearance potentials of NF_2_, NF_3_, the *cis* and *trans* isomers of N_2_F_2_, and N_2_F_4_ were measured as described in previous work [7]. For NF_2_, measurements were made on the vapors effusing from the reactor containing N_2_F_4_, at 170 °C.

The NF_3_ and N_2_F_4_ were obtained through D. E. Mann. Their purity has been noted elsewhere [1, 3]. The *cis* and *trans* isomers of N_2_F_2_ were kindly prepared and purified for us by Charles S. Cleaver of the E. I. Du Pont de Nemours Experimental Station, Wilmington, Del. Immediately after separation by gas chromatography, the isomers were placed in Monel cylinders and cooled with solid CO_2_. They were transported and maintained at this temperature until introduced to the mass spectrometer. Gas chromatographic analysis reported by Cleaver indicated the following compositions:

*trans*–N_2_F_2_: 0.2% air, <0.1% NF_3_, <0.1% N_2_O,>99.6% *trans*–N_2_F_2_;
*cis*–N_2_F_2_: 0.6% air, 0.2% N_2_O, 5.2% *trans*–N_2_F_2_, 94.0%. *cis*–N_2_F_2_.

These analyses were supported by our mass spectrometric observations.

For conversion from electron volts to joules, 1 ev is taken to be 9.6496×10^4^ joules. For conversion to the thermochemical calories, 1 cal is taken to be 4.1840 joules.

## 3. Results and Discussion

### 3.1. Thermal Dissociation of N_2_F_4_

A typical set of data for the thermal dissociation of N_2_F_4_ is summarized in table 1. Column 1 gives the absolute temperature of the reactor, and columns 2 and 3 the observed ion currents of the N_2_F_4_^+^ and NF_2_^+^ ions in arbitrary units.

For a first approximation, it is assumed that no NF_2_ is formed at the lowest reactor temperature, i.e., 333.0 °K. The ratio of NF_2_^+^/N_2_F_4_^+^ at this temperature was taken as characteristic of the mass spectrum of N_2_F_4_ and was applied to the data in column 2, table 1 to calculate the contribution to the observed NF_2_^+^ peak of NF_2_^+^ ions resulting from dissociative ionization of N_2_F_4_ (column 4). The contribution resulting from the ionization of NF_2_ is obtained by difference (column 5). On the further assumption that the observed N_2_F_4_^+^ ion abundance and the calculated NF_2_^+^ ion abundance are measures of the partial pressures of N_2_F_4_ and NF_2_, respectively, an equilibrium constant can be obtained from the relation

$$K_{p}=k{\left({\text{NF}}_{2}\right)}^{2}/N_{2}F_{4}$$ (1)

where *k* is a factor relating measured ion abundances to partial pressures. Values of *K_p_*/*k* are given in column 6.

**Figure 2:**
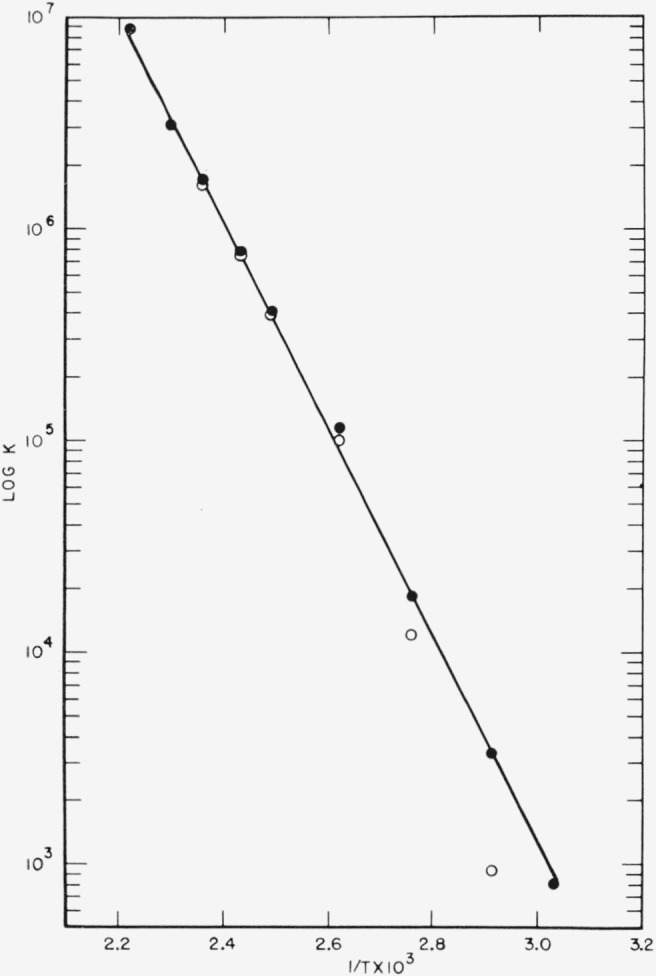
Log *K_p_* versus 1/*T* for the equilibrium *N*_2_*F*_4_⇌2*NF*_2_.

From the usual integrated van’t Hoff equation, we plot log *K_p_* versus 1/*T* to obtain the enthalpy, Δ*H*, of the reaction. In this case, however, the slope of the plot must be obtained by successive approximation. The data of table 1 are plotted as the open circles of figure 2. The best straight line through these points is extrapolated to the lowest temperature (333.0 °K) and a first estimate made of the ratio NF_2_/N_2_F_4_ from eq (1). This is then used to calculate a more nearly correct set of data. The process is repeated until the indicated constant slope is obtained, shown as solid circles in figure 2. The mean of four such determinations, resulted in a value of Δ*H*=5.14 ±0.38 kj/mole(21.5± 1.6 kcal/mole). The uncertainty given is the estimated standard deviation. The value of the gas constant used in the calculations was *R*=8.314 joule/degree mole. This is in good agreement with the previously reported value of 19.2 kcal/mole [4].

From the value, Δ*H*=21.5 ±1.6 kcal/mole for the reaction N_2_F_4_→2NF_2_, and the Δ*H_f_*(N_2_F_4_) = −2.0±2.5 kcal/mole [8] we calculate Δ*H_f_*(NF_2_)=9.8±2.1 kcal/mole. Further, from Δ*H_f_*(NF_3_) = −29.7 ±1.8 kcal/mole [9] and Δ*H_f_*(F) = 18.9±0.5 kcal/mole [10], we calculate D(NF_2_-F) = 58. 4±4.4 kcal/mole. Similarly, from NF_2_→N+2F, we calculate D(N–F) average, in NF_2_=70.5±1.6 kcal/mole. Finally, from NF_2_→NF+F, we calculate Δ*H_f_* (NF) = 61.4 ±4.2 kcal/mole.

As the average bond energy in NF_3_ is 66.3 kcal/mole [9], it would appear that the first N—F bond is the weakest bond in NF_3_. This is contrary to the observed bond order in NH_3_, in which the first and subsequent N—H bond dissociation energies are reported to be 104, 88, and 88 kcal/mole, respectively [11]. This would negate the assumptions made by Reese and Dibeler [1] in their calculations of the ionization potentials of NF_2_ and NF radicals.

### 3.2 Appearance Potential Data

Two studies of N_2_F_4_ have been reported [2,3] but the original interpretation of the NF^+^ and NF_2_^+^ appearance potentials did not account for the dissociation of N_2_F_4_ into NF_2_ radicals within the ion source.

**Figure 3:**
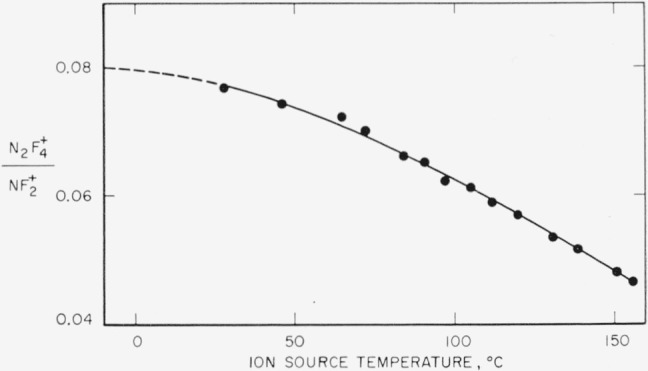
Effect of ion source temperature on the *N*_2_*F*_4_^+^/*NF*_2_^+^ ratio in the mass specturm of *N*_2_*F*_4_.

The effect of ion source temperature on the N_2_F_4_^+^/NF_2_^+^ ratio in the mass spectrum of N_2_F_4_ is shown in figure 3. Although an extrapolation of the data to lower temperatures is difficult, it seems apparent that the limiting value of the ratio is about 0.08. The change in mass spectrum of N_2_F_4_ with temperature, due to decomposition of N_2_F_4_ in the ion source, thus accounts for the differences in the mass spectrum of N_2_F_4_ reported by different workers [2, 3, 4, 12]. The data of Loughran and Mader [2] have already been reinterpreted assuming the presence of NF_2_ [4] in the ion source.

A summary of the available appearance potential data for the N—F compounds is shown in table 2. Column 1 identifies the molecule, columns 2 and 3 give the ion and the probable process of formation, column 4 gives the observed appearance potential and column 5 reports the source.

#### NF_2_

The ionization potential of NF_2_ measured in this work was 12.0 ±0.1 ev in good agreement with that of Loughran and Mader. The average of the two values is 11.9 ±0.2 ev.

Differences in the reported NF^+^ appearance potentials from NF_2_ are much greater. We observe two processes leading to the formation of NF^+^. The difference in the appearance potentials of these processes is almost equal to the electron affinity of the fluorine atom (3.6 ev) [13]. This gives considerable support to the present identification.

From

$$\begin{array}{c} {\text{NF}}_{2}\to {\text{NF}}^{+}+F\\ A\left({\text{NF}}^{+}\right)\geq D\left(\text{NF}-F\right)+I\left(\text{NF}\right) \end{array}$$

where the inequality accounts for any excess energy involved in the reaction, we calculate an upper limit for I(NF) = 12.4±0.3 ev, assuming D(NF−F) = D(N−F) average in NF_2_. This differs from the previous estimate of I(NF) = 12.0 ev [1]. However, the present value is considered the more reliable for reasons stated in the previous section.

#### NF_3_

The two reported values for the appearance potential of the NF_2_^+^ ion from NF_3_ differ by 0.4 ev. Different methods of evaluating the appearance potential were used by each investigator. We also find it possible, by using different graphical methods, to interpret our data so as to obtain either limiting value from the same set of measurements. However, the appearance potential is readily calculated from the equation

$${\text{NF}}_{3}\to {{\text{NF}}_{2}}^{+}+F$$

from which

$$\begin{array}{c} A\left({{\text{NF}}_{2}}^{+}\right)\geq D\left({\text{NF}}_{2}-F\right)+I\left({\text{NF}}_{2}\right)\\ \geq 14.4\pm 0.4\;\text{ev}. \end{array}$$

The calculated value lies just between the two limiting experimental values.

The NF^+^ appearance potential has been reported as 17.9±0.3 ev [1], and ascribed to the reaction

$${\text{NF}}_{3}\to {\text{NF}}^{+}+2F.$$

From the relation

$${A(\text{NF}}^{+})\geq D\left({\text{NF}}_{2}-F\right)+D\left(\text{NF}-F\right)+I\left(\text{NF}\right)$$

and the values of D(NF_2_–F), D(NF–F), and I(NF) given above, we calculate A(NF^+^)≥18.0 ±0.6 ev, in good agreement with the measured value. Thus there appears to be no evidence for a lower energy process for this reaction which would result in the formation of molecular fluorine.

#### N_2_F_2_

The mass spectra of the *cis* and *trans* N_2_F_2_ were similar in most respects to those reported previously [5,13], However, additional very diffuse peaks in the mass spectra at nonintegral m/e ratios were observed and attributed to metastable transitions [15]. These metastable ions were observed only in the mass spectrum of the *trans* species. This is consistent with the fact that the *cis* isomer apparently produces no parent ion. The relative abundance of the metastable ion appearing at the nominal m/e=33.5 was 0.22 percent of the largest normal ion peak and was attributed to the transition, N_2_F_2_^+^→N_2_F^+^+F. The ion appearing at m/e=16.5 was 0.02 percent of the maximum peak and was attributed to the transition, N_2_F_2_^+^→NF^+^+NF. Appearance potential measurements of the ions at m/e=33.5 and 16.5 ruled out the possibility of doubly charged ions.

The relatively large abundance of the m/e=33.5 metastable peak in *trans* N_2_F_2_ made it possible to measure the appearance potential of this ion with good precision. As might be expected on the basis of the statistical theory of mass spectra [12], the appearance potential is somewhat lower than that of the same ions collected at m/e=47. However, the magnitude of the difference is unexpectedly large.

The appearance potentials of the normal fragment ions NF^+^ and N_2_F^+^ are identical within experimental uncertainty for both *cis* and *trans* N_2_F_2_. The heats of formation of the two isomers are also very similar; thus Armstrong and Marantz [16] report Δ*H_f_*(N_2_F_2_) *cis*=16.4 kcal/mole and Δ(N_2_F_2_) *trans*=19.4 kcal/mole with an uncertainty of about 1.5 kcal/mole. Thus if there is no excess kinetic or excitational energy involved in the dissociative ionization of either of the isomers, it would appear that they are similar in molecular structure.

This argues in favor of the *cis* and *trans* designations for the N_2_F_2_ isomers contrary to the recent suggestion by Sanborn [5] that the isomer presently designated “*cis*” actually has the 1,1-difluorodiazine structure as first considered by Bauer [17].

Similarly, these data do not support the recently reported [18] heat of isomerization of 27.5±5.0 kcal/mole for the N_2_F_2_ isomers. However, we have been unable to calculate this value from the data as given in the reference.

On the basis of nearly equal heats of formation for the *cis* and *trans* isomers, we can calculate the N = N bond dissociation energy for either isomer of N_2_F_2_ from the reaction:

$$N_{2}F_{2}\to {\text{NF}}^{+}+\text{NF}$$

and the relation D (FN=NF) ≤ A(NF^+^) − I (NF). Using the values A(NF^+^) = 17.0±0.2 ev and I(NF) = 12.4±0.3 ev, we obtain D(FN=NF)≤4.6±0.5 ev, or ≤ 106 ±12 kcal/mole.

A check on this calculation can be made using the measured values for Δ*H_f_* (N_2_F_2_) and the reaction

$$N_{2}F_{2}\to 2\text{NF}$$

from which D (FN=NF)=2Δ*H_f_*/NF−Δ*H_f_*N_2_F_2_. Using the previously calculated value for Δ*H_f_*NF = 64.4 ±4.2, we calculate D(FN=FN) *cis*=106±10 kcal/mole and D (FN=NF) *trans*= 103 ± 10 kcal/mole.

These values may be compared with the value of D(HN = NH) = 104±6 kcal/mole in diimide as reported by Foner and Hudson [19]. However, it should be emphasized that both methods used to calculate D(FN=NF) involve a common approximation, i.e., that the bond dissociation energy D(FN−F) = D(N−F) average in NF_2_. The uncertainty in these and previous calculations are conservatively estimated from the algebraic sum of uncertainties in the contributing measurements.

A summary of measured and derived thermochemical data for the N−F compounds is given in table 3.

## Figures and Tables

**Table 1:** Summary—calculation of the equilibrium constants for the thermal dissociation of *N*_2_*F*_4_

*T*(°K)	Observed ion currents		${(\text{NF}}_{2}^{+}{)N}_{2}F_{4}$	${(\text{NF}}_{2}^{+}{)\text{NF}}_{2}$	$\frac{K_{p}}{k}=\frac{{\left({\text{NF}}_{2}\right)}^{2}}{N_{2}F_{4}}$	${(\text{NF}}_{2}^{+}{)N}_{2}F_{4}$	${(\text{NF}}_{2}^{+}{)\text{NF}}_{2}$	$\frac{K_{p}}{k}=\frac{{\left({\text{NF}}_{2}\right)}^{2}}{N_{2}F_{4}}$	${(\text{NF}}_{2}^{+}{)N}_{2}F_{4}$	${(\text{NF}}_{2}^{+}{)\text{NF}}_{2}$	$\frac{K_{p}}{k}=\frac{{\left({\text{NF}}_{2}\right)}^{2}}{N_{2}F_{4}}$
	N_2_F_4_^+^	NF_2_^+^									
											
450.7	28.0	16320	660	15660	8.76×10^6^	640	15680	8.78×10^6^	630	15690	8.79×10^6^
434.3	68.5	16140	1620	14520	3.09	1260	14580	3.11	1550	14590	3.11
423.9	111.0	16110	2620	13490	1.64	2540	13570	1.66	2510	13600	1.67
412.0	184	16110	4350	11760	7.50×10^5^	4200	11910	7.72×10^5^	4160	11950	7.76×10^3^
401.6	259	16200	6110	10090	3.92	5920	10280	4.08	5900	10300	4.10
382.4	440	17070	10400	6670	1.01	10040	7030	1.12	9950	7120	1.15
362.0	650	18150	15330	2820	1.22×10^4^	14840	3310	1.69×10^4^	14700	3450	1.83×10^4^
343.6	740	18300	17470	830	9.32×10^2^	16900	1400	2.65×10^3^	16720	1580	3.37×10^3^
333.0	750	17700	17700	0	0	17120	580	4.49×10^2^	16920	775	8.01×10^2^

**Table 2:** Summary of appearance potential data for *N*–*F* compounds

Parent molecule	Ion	Probable process	Appearance potential	Reference
				
			*ev*	
NF_2_	$\{\begin{array}{l} {{\text{NF}}_{2}}^{+}\\ {\text{NF}}^{+} \end{array}$	NF_2_→NF_2_^+^	$\{\;\begin{array}{l} 12.0\pm 0.1\\ 11.8\pm 0.2 \end{array}$ 11.8±0.2	This work. [2] This work.
		NF_2_→NF^+^+F^−^→NF^+^+F	$\{\;\begin{array}{l} 15.5\pm 0.2\\ 15.0\pm 0.2 \end{array}$	Do. [2]
NF_3_	$\{\begin{array}{l} {{\text{NF}}_{3}}^{+}\\ {{\text{NF}}_{2}}^{+}\\ {\text{NF}}^{+} \end{array}$	NF_3_→NF_3_^+^	$\{\;\begin{array}{l} 13.2\pm 0.2\\ 13.2 \end{array}$^a^	[1] This work.
		NF_3_→NF_2_^+^+F	$\{\;\begin{array}{l} 14.2\pm 0.3\\ 14.6\\ 14.2\to 14.6 \end{array}$^a^	[1] [2] This work.
		NF_3_→NF^+^+2F	17.9±0.3	[1]
*trans*-N_2_F_2_	$\{\begin{array}{l} N_{2}{F_{2}}^{+}\\ N_{2}F^{+}\\ {\text{NF}}^{+} \end{array}$	N_2_F_2_→N_2_F_2_^+^	13.1±0.1	This work.
		N_2_F_2_→N_2_F^+^+F	13.9±0.2	Do.
		N_2_F_2_^+^→N_2_F^+^+F (metastable)	13.4±0.2	Do.
		N_2_F_2_→NF^+^+NF	17.0±0.2	Do.
*cis*-N_2_F_2_	$\{\begin{array}{l} N_{2}F^{+}\\ {\text{NF}}^{+} \end{array}$	N_2_F_2_→N_2_F^+^+F	14.0±0.2	Do.
		N_2_F_2_→NF^+^+NF	16.9±0.2	Do.
N_2_F_4_	$\{\begin{array}{l} N_{2}{F_{4}}^{+}\\ N_{2}{F_{3}}^{+}\\ {{\text{NF}}_{2}}^{+}\\ {\text{NF}}^{+} \end{array}$	N_2_F_4_→N_2_F_4_+	12.0±0.1	[3]
		→N_2_F_3_^+^+F^−^	12.0^a^	This work.
		→N_2_F_3_^+^+F^−^	15.6^a^	Do.
		See text.		
		See text.		

a Single observation.

**Table 3:** Summary of thermochemical data for *N*—*F* compounds

Molecule	Δ*H_f_*	Ionization potential	Bond dissociation energy
			
	*kcal*/*mole*	*ev*	*kcal*/*mole*
NF	61.4±4.2	≤12.4±0.3	……………………………
NF_2_	9.8±2.1	12.0±0.1	D(N−F)av=70.5±1.6
NF_3_	−29.7±1.8[9]	13.2±0.2[1]	D(F_2_N−F) = 58.4±4.4
*cis* N_2_F_2_	16.4±1.5[16]		D(FN = NF)=106±10
*trans* N_2_F_2_	19.4±1.5[16]	13.1±0.1	D(FN=NF) =103±10
N_2_F_4_	−2.0±2.5[8]	12.0±0.1 [3]	D(F_2_N−NF_2_)=21.5±1.6

## Footnotes

1 Figures in brackets indicate the literature references at the end of this paper.
